# Preclinical Evaluation of a Novel Dual Targeting PI3Kδ/BRD4 Inhibitor, SF2535, in B-Cell Acute Lymphoblastic Leukemia

**DOI:** 10.3389/fonc.2021.766888

**Published:** 2021-12-01

**Authors:** Yongsheng Ruan, Hye Na Kim, Heather A. Ogana, Zesheng Wan, Samantha Hurwitz, Cydney Nichols, Nour Abdel-Azim, Ariana Coba, Seyoung Seo, Yong-Hwee Eddie Loh, Eun Ji Gang, Hisham Abdel-Azim, Chih-Lin Hsieh, Michael R. Lieber, Chintan Parekh, Dhananjaya Pal, Deepa Bhojwani, Donald L. Durden, Yong-Mi Kim

**Affiliations:** ^1^ Department of Pediatrics, Division of Hematology, Oncology, Blood and Marrow Transplantation, Children’s Hospital Los Angeles, Norris Comprehensive Cancer Center, University of Southern California Keck School of Medicine, Los Angeles, CA, United States; ^2^ Department of Pediatrics, Nanfang Hospital, Southern Medical University, Guangzhou, China; ^3^ University of Southern California (USC) Libraries Bioinformatics Services, University of Southern California, Los Angeles, CA, United States; ^4^ University of Southern California (USC) Department of Urology, University of Southern California (USC) Norris Comprehensive Cancer Center, Los Angeles, CA, United States; ^5^ University of Southern California (USC) Department of Pathology, University of Southern California (USC) Norris Comprehensive Cancer Center, Los Angeles, CA, United States; ^6^ Department of Pediatrics, University of California San Diego, San Diego, CA, United States; ^7^ SignalRx Pharmaceuticals Inc., Omaha, NE, United States

**Keywords:** PI3Kδ, p-AKT, BRD4, c-Myc, acute lymphoblastic leukemia, SF2535

## Abstract

The PI3K/Akt pathway—and in particular PI3Kδ—is known for its role in drug resistant B-cell acute lymphoblastic leukemia (B-ALL) and it is often upregulated in refractory or relapsed B-ALL. Myc proteins are transcription factors responsible for transcribing pro-proliferative genes and c-Myc is often overexpressed in cancers. The chromatin regulator BRD4 is required for expression of c-Myc in hematologic malignancies including B-ALL. Previously, combination of BRD4 and PI3K inhibition with SF2523 was shown to successfully decrease Myc expression. However, the underlying mechanism and effect of dual inhibition of PI3Kδ/BRD4 in B-ALL remains unknown. To study this, we utilized SF2535, a novel small molecule dual inhibitor which can specifically target the PI3Kδ isoform and BRD4. We treated primary B-ALL cells with various concentrations of SF2535 and studied its effect on specific pharmacological on-target mechanisms such as apoptosis, cell cycle, cell proliferation, and adhesion molecules expression using*in vitro* and *in vivo* models. SF2535 significantly downregulates both c-Myc mRNA and protein expression through inhibition of BRD4 at the c-Myc promoter site and decreases p-AKT expression through inhibition of the PI3Kδ/AKT pathway. SF2535 induced apoptosis in B-ALL by downregulation of BCL-2 and increased cleavage of caspase-3, caspase-7, and PARP. Moreover, SF2535 induced cell cycle arrest and decreased cell counts in B-ALL. Interestingly, SF2535 decreased the mean fluorescence intensity (MFI) of integrin α4, α5, α6, and β1 while increasing MFI of CXCR4, indicating that SF2535 may work through inside-out signaling of integrins. Taken together, our data provide a rationale for the clinical evaluation of targeting PI3Kδ/BRD4 in refractory or relapsed B-ALL using SF2535.

## Introduction

Despite a high five-year survival rate, relapsed and refractory B-cell acute lymphoblastic leukemia (B-ALL) remains a problem in children (1) the and prognosis for adult B-ALL patients is poor (2). During treatment, leukemia cells interact with the bone marrow (BM) microenvironment and obtain a survival benefit, known as cell adhesion-mediated drug resistance (CAM-DR) (3). This drug resistance in B-ALL can be achieved by increased pro-survival intracellular signaling as a result of adhesion to the BM microenvironment. The PI3K-AKT pathway has been identified as one of the most significant pro-survival pathways in CAM-DR and leukemia cell-BM stromal cell contact has been shown to upregulate phosphorylated AKT in B-ALL (4). Despite great interest in inhibition of the AKT pathway *via* targeting PI3K isoforms in leukemia, a clinically available drug for B-ALL treatment remains elusive (4–6).

In addition, PI3K inhibition facilitates degradation of the transcription factor MYC through the GSK-3β-dependent MYC phosphorylation pathway (7). Emerging reports have indicated oncogenic protein c-Myc plays a critical role in survival, proliferation, and drug resistance in both B and T-ALL (8–11). However, direct targeting of Myc has been a challenge due to its “undruggable” protein structure (12). Currently, targeting c-Myc transcription by interfering with chromatin-dependent signal transduction to RNA polymerase by BRD4 inhibition has shown great promise (12, 13). BRD4 is a member of the bromodomain and extraterminal domain (BET) family of proteins which binds to acetylated lysine residues at promoter and enhancer regions, including regions for the *MYC* gene (14). BRD4 has been proposed to be a critical chromatin regulator that maintains disease progression in acute myeloid leukemia (AML) (15). As a result, suppression of BRD4 with shRNA or JQ1, a bromodomain inhibitor, caused anti-leukemic effects *in vitro* and *in vivo.* An increasing number of studies show promising results of BET protein inhibition with preclinical inhibitors, such as JQ1 in AML cell lines, *ex vivo* patient samples, or mouse models (16, 17). BET inhibition also been shown to be efficient against primary childhood B-ALL by decreasing c-Myc protein stability, suppressing progression at DNA replication forks, and sensitizing primary B-ALL towards dexamethasone *in vitro* and *in vivo* (18). There are few BET inhibitors that have been used in clinical trials, including OTX015 (MK-8628), an analog of JQ1, in a Phase 1 trial for AML (19). In this dose-escalation study, three patients achieved complete remission and two additional patients had partial blast clearance (19). Previous studies have shown that concomitant inhibition of PI3K and BRD4 by SF2523 blocks MYC expression and activation, promotes MYC degradation, and markedly inhibits neuroblastoma cell growth and metastasis (20). Taken together, PI3K and BRD4 inhibition cause downregulation of c-Myc owing to promotion of c-Myc degradation and attenuation of c-Myc transcription. Therefore, it is a rationale for synthesis of a dual targeting PI3K and BRD4 inhibitor (21, 22).

Herein, we evaluated SF2535, a novel small molecule inhibitor of PI3Kδ and BRD4, in B-ALL. We have reported the chemical structures of SF2535, which is a derivative of SF2523 (20). Both SF2535 and SF2523 were found from a discovery of the 5-morpholino-7H-thieno[3,2-b]pyran-7-one (TP-scaffold) system, which was the foundation of a new compound class of potential PI3K inhibitors with improved potency. As BRD4 bromodomains (BDs) are targets of TP-scaffold inhibitors, both SF2535 and SF2523 bind to BRD4 BD1 to a similar extent according to displacement and NMR titration experiments (20). Unlike SF2523, which is a highly selective and potent inhibitor of PI3K, particularly of the PI3Kα isoform, SF2535 specifically targets PI3Kδ. Since the PI3Kδ isoform is expressed selectively in hematopoietic cells and PI3Kδ signaling is active in many B-cell leukemias and lymphomas (23), we chose B-ALL as the disease model for the preclinical evaluation of SF2535.

## Materials and Methods

### Patient Samples and Cell Culture

Bone marrow samples were obtained from B-ALL patients after informed signed consent from patients in compliance with the Institutional Review Board regulations of Children’s Hospital Los Angeles. Primary B-ALL blasts from bone marrow aspirates were isolated by Ficoll (GE Healthcare) gradient centrifugation and co-cultured with irradiated OP9 stroma cells (ATCC) in MEM-alpha supplemented with 20% fetal bovine serum (FBS, Invitrogen), 100U/ml penicillin and 100µg/ml streptomycin at 37°C and 5% CO_2_. Patient sample information is listed in **Table S1**.

### Starvation and Activation Assay for Detection of Phosphorylated-Akt^Ser473^

B-ALL cells were serum-deprived by washing twice with Dulbecco’s Phosphate-Buffered Saline (DPBS, Invitrogen) and cultured in MEM-alpha media at 37°C and 5% CO_2_ overnight. Following another wash with DPBS, B-ALL cells were treated with vehicle control DMSO or SF2535 for 30 minutes. Subsequently, FBS was added to a final concentration of 20% to all cells except for the no-activation control groups. Whole cell lysates were isolated after 1 hour for Western blot analysis for phosphorylated-Akt^Ser473^ (p-AKT^S473^) detection.

### Western Blot

B-ALL cells were harvested and lysed in M-PER buffer (Invitrogen) containing 1% protease inhibitor cocktail (VWR). Protein concentration was determined by Bradford protein assay. Proteins were separated by 4-12% Bis-Tris protein gels (Invitrogen) and transferred to PVDF membranes (Invitrogen). The antibodies (Abs) used are listed in **Table S2**.

### Chromatin Immuno-Precipitation (ChIP)

Cells were treated with DMSO or SF2535 5µM for 18 hours and were then harvested and processed using ChIP kit according to the manufacturer instructions (Abcam). In brief, the cells were fixed with 1.1% Formaldehyde, quenched by 10% glycine, and lysed. The lysates were sonicated in order to shear DNA to form DNA fragments with optimal size of 200-1000bp. A portion of the diluted chromatin was set aside for the INPUT. Diluted chromatins were incubated with anti-BRD4 (1:50, CST), anti-histone H3 (4µg, Abcam) as positive control, or no antibody as negative control overnight with rotation at 4°C. The antibody binding beads were added and washed according to the manufacturer’s instructions. The samples were treated with DNA-purifying slurry and Proteinase K to purify DNA. Samples were subjected to qPCR using the c-MYC promoter primers (F: 5’-GAGCAGCAGAGAAAGGGAGA-3’, R: 5’-CAGCCGAGCACTCTAGCTCT-3’). Fold enrichment was analyzed as described previously described (24).

### RNA Extraction and qPCR

Cells were treated with DMSO or 5µM SF2535 for 6 hours. Total RNA was extracted using the Qiagen RNeasy kit (Qiagen) and cDNA was produced by the SuperScript III First-Strand Synthesis System (Invitrogen). cDNA was amplified by specific c-Myc primers (F 5’-CTTCTCTCCGTCCTCGGATTCT-3’; R 5’-GAAGGTGATCCAGACTCTGACCTT-3’) and GAPDH primers (F 5’-GTTGCCATCAATGACCCCTTCATTG-3’; R 5’-GCTTCACCACCTTCTTGATGTCATC-3’) with PowerUp SYBR Green Master Mix (Applied Biosystems) using a ABI 7900HT qPCR machine. Relative expression levels of c-Myc were normalized to GAPDH expression and calculated as described previously (24).

### Apoptosis Analysis With Annexin V and DAPI Staining

Following 24 hours or 72 hours treatment of DMSO or SF2535, B-ALL cells were resuspended in 1X Annexin V binding buffer (Becton Dickinson) at a concentration of 1×10^6^ cells per mL. 2.5μl Annexin V PE (BioLegend) and 2.5μl DAPI (50μg/mL, Invitrogen) were added to 100μl of the cell suspension. After 15 min incubation at room temperature in the dark, B-ALL cells were analyzed by flow cytometry using BD FACS Canto II.

### Cell Cycle Analysis

B-ALL cells were treated with DMSO or SF2535 (0.2µM 1µM, or 5µM) for 24 hours. Subsequently, cells were stained with CytoPhase™ Violet (BioLegend) at 5µM and incubated for 90 minutes at 37°C and 5% CO_2_ and analyzed on a BD FACSCanto II flow cytometer. Furthermore, BrdU incorporation assay (Phase-Flow™ BrdU cell proliferation kit FITC-conjugated, BioLegend) according to the protocol of the manufacturer was performed as confirmation of the results. In brief, BrdU solution was added to cell suspension at 0.5μL/mL. Following 1.5-hour incubation, B-ALL cells were harvested and washed. Buffer A was added for 20 minutes at 4°C to fix cells. Then after cell permeabilization and repeat fixation of cells, cells were treated with DNAse and incubated 1 hour at 37°C. Lastly, 5μL of anti-BrdU antibody was added to each tube for 15 minutes at room temperature in the dark. Cells were resuspended with PBS containing DAPI (1μg/mL) prior to acquiring on a flow cytometer.

### Cell Proliferation Assay

1×10^6^ B-ALL cells were seeded per condition in triplicates on irradiated OP9 stromal cells as previously described (25). B-ALL cells were treated with DMSO or SF2535 (0.2µM 1µM, or 5µM) for 24 hours and 72 hours. Cell numbers were counted by Trypan blue exclusion on a hemocytometer under an inverted phase-contrast microscope.

### Flow Cytometry

B-ALL cells were treated with the indicated concentration of SF2535 for 24h. Subsequently, B-ALL cells were resuspended in 100µl PBS containing FACS antibodies or the respective isotype controls (information can be found in **Table S3**). Following incubation at 4°C for 30 min, B-ALL cells were washed by 1ml PBS and resuspended in PBS containing DAPI (1μg/mL) then analyzed with a BD FACSCanto II flow cytometer. Flow cytometry data was analyzed with FlowJo 7.0 software (FlowJo LLC).

### Cell Adhesion Assay

2.5 × 10^4^/well irradiated OP9 cells were seeded onto a tissue culture 96-well plates and cultured overnight. Simultaneously, B-ALL cells were treated with different concentrations of SF2535 or DMSO for 24 hours. Then live B-ALL cells were harvested, washed once with DPBS, and resuspended at the final concentration of 0.2 × 10^6^/200µL with culture medium. B-ALL cells were dispended onto an irradiated OP9 96-well plate with 200 µL in each well and allowed to adhere for 2 hours at 37°C. Non-adhering cells were removed and remaining cells on OP9 were gently washed with 100 µL of DPBS. Adherent cells and supernatant cells were counted by Trypan blue exclusion on a hemocytometer.

### Animal Studies

Primary relapsed B-ALL cells (LAX56) were intravenously injected into NOD.Cg-*Prkdc^scid^ Il2rg^tm1Wjl^*/SzJ (NSG, The Jackson Laboratory) mice (1×10^6^cells/mouse). After 3 weeks of engraftment, SF2535 (30 mg/kg, dissolved in 20% dimethylacetamide (DMA) and 80% Captisol (20% w/v in water for 2mg/ml)) (n=6) or vehicle (n=6) was administered once by intraperitoneal (i.p.) injection. After 24 hours, mice were sacrificed and bone marrow, spleen, and peripheral blood were harvested, and red blood cells were lysed by RBC lysis buffer (Invitrogen). The animal study was performed in compliance with a research protocol approved by the Institutional Animal Care and Use Committee (IACUC), the Saban Research Institute of Children’s Hospital Los Angeles.

### Data Analysis and Statistics

All statistical analyses were performed using GraphPad Prism 5. The mean was chosen as a center value for all graphs. 95% confidence interval (95% CI), a standard deviation of the mean was used as measures of spread as indicated in figure legends and the *Results* section. Statistical analysis was performed using paired Student’s t-test or one-way ANOVA followed by Tukey’s multiple comparison tests for statistical analyses as appropriate. A p-value of <0.05 was considered statistically significant.

### Treatment of Normal B Cells With SF2535

0.5x10^6^ of immortalized EBV-transformed normal B cell lines 3301015 and 5680001 (kind gift from Dr. Hsieh and Dr. Lieber) were treated with 5μM of SF2535 in 1mL of R10 medium in a 24-well tissue culture plate. After 24hours, cells were harvested and washed with DPBS. Washed cells were stained with 7AAD and Annexin V in Annexin V staining buffer. Viability of cells was assessed by measuring the percentage of 7AAD/Annexin V double negative population.

## Results

### PI3Kδ and BRD4 Expression in B-ALL

**Figure 1:**
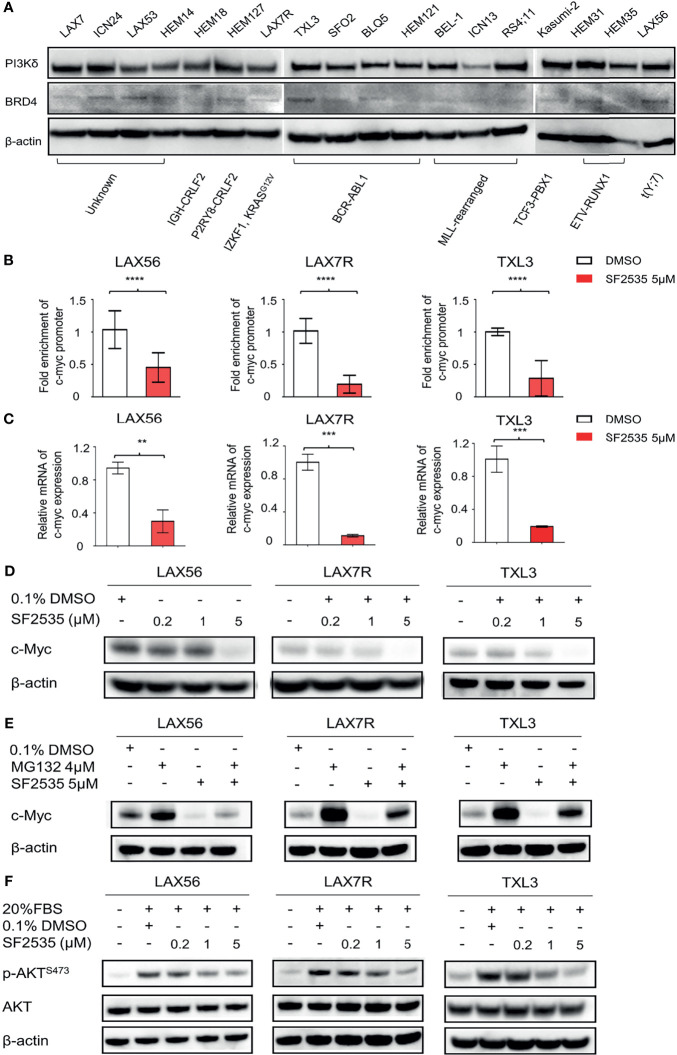
SF2535 downregulates c-Myc and p-AKT. **(A)** PI3Kδ and BRD4 expression in whole cell lysates in B-ALL. **(B)** Primary B-ALL LAX56, LAX7R and TXL3 cells were treated with SF2535 at 5µM. After 18 hours, the cells were harvested for BRD4 ChIP analysis which was performed at the c-Myc promoter site. Data were combined from three independent experiments per leukemia. Data was analyzed by paired Student’s t-test, where ****P<0.0001 vs. ctrl (DMSO). **(C)** qPCR data showing the effect of SF2535 on c-Myc expression in B-ALL cells. Experiment was performed in triplicate. Data was analyzed by Student’s t test, where **P<0.01, ***P<0.001 vs. ctrl (DMSO). **(D)** LAX56, LAX7R and TXL3 cells were treated with 0.1% DMSO or SF2535 (0.2μM, 1μM, or 5μM) for 48 hours. c-Myc expression of B-ALL cells was analyzed by Western blot. **(E)** LAX56, LAX7R, and TXL3 cells were pre-treated with either 0.1%DMSO control or proteasome inhibitor MG132 (4μM) for 45 min and subsequently treated with SF2535 at 5μM for 6 hours. c-Myc expression was analyzed by Western blot. **(F)** LAX56, LAX7R and TXL3 cells were cultured in MEM-α without serum overnight. Subsequently, cells were treated with 0.1% DMSO control, SF2535 (0.2μM, 1μM, 5μM) for 30 mins. Cells were activated with 20% FBS for 1 hour. Western blots of p-AKT^S473^ and AKT are shown. β-actin was used as internal control for equal protein loading for Western blots **(D–F)**. One of two independent experiments per leukemia was performed for **(D–F)**.

Expression of PI3Kδ and BRD4 was determined in fifteen primary B-ALL and three B-ALL cell lines representing various cytogenetics (**Table S1**). Most of the primary B-ALL and cell lines expressed similar levels of PI3Kδ despite their difference in cytogenetics, while BRD4 levels were variable (**Figure 1A**). In order to choose primary B-ALL patient samples for further analysis, we made selections based on their diagnosis status (relapsed or refractory), expression of both PI3Kδ (**Figure S1A**) and BRD4 (**Figure S1B**) based on quantitative densitometric analysis, and inclusion of a wide range of karyotypes including *BCR-ABL1*+ status. Based on these factors we selected LAX56, LAX7R, and TXL3 for subsequent studies.

### SF2535 Downregulates c-Myc and p-AKT in B-ALL

To determine the effective concentration of SF2535 in primary B-ALL cases, we treated three primary B-ALL (LAX56, LAX7R, and TXL3) cells with increasing concentrations of SF2535 for 48 hours. SF2535 dose-dependently induced apoptosis in all B-ALL cells, and the calculated EC_50_ values of SF2535 were 2.4μM (95% CI, 1.990μM - 2.935μM), 1.5μM (95% CI, 1.389μM - 1.633μM), and 3.2 μM (95% CI, 2.718μM – 3.670μM) in LAX56, LAX7R and TXL3, respectively (**Figure S2**). Based on these values, three different doses of SF2535, 0.2μM, 1μM, and 5μM, were chosen for subsequent studies. As c-Myc transcription is mediated by BRD4 binding to the promoter region (12), we evaluated the specific effect of SF2535 on BRD4 binding on the c-Myc promoter by chromatin immunoprecipitation (ChIP). In all three cases, SF2535 decreased BRD4 binding to the c-Myc promoter site compared to DMSO (LAX56 P<0.0001, LAX7R P<0.0001, TXL3 P<0.0001) (**Figure 1B**). Subsequently, mRNA transcript levels of c-Myc expression were significantly decreased upon SF2535 treatment in all three cases (LAX56 P=0.0020, LAX7R P=0.0002, TXL3 P=0.0009) (**Figure 1C**). Finally, SF2535 prominently downregulated c-Myc protein expression in a dose-dependent manner, which was determined by Western blot (**Figure 1D**, **Figure S3A**). Decrease in c-Myc protein expression could be restored by the proteasome inhibitor MG132, which shows c-Myc degradation in B-ALL occurs through the ubiquitin-proteasome pathway (26) (**Figure 1E**, **Figure S3B**). SF2535 also decreased phosphorylated AKT in LAX56, LAX7R and TXL3, demonstrating the on-target effect of SF2535 on PI3Kδ. Following serum starvation of leukemia cells, B-ALL cells were treated with DMSO (vehicle control) or 0.2μM, 1μM, or 5μM of SF2535 for 30 minutes followed by 1 hour serum-induced activation. Levels of p-AKT^S473^ decreased in a dose-dependent manner from SF2535 in LAX56, LAX7R, and TXL3 B-ALL cells (**Figure 1F**, **Figures S3C, D**).

### SF2535 Induces Apoptosis in B-ALL Cells Through Changes in the Intrinsic Apoptotic Pathway

**Figure 2:**
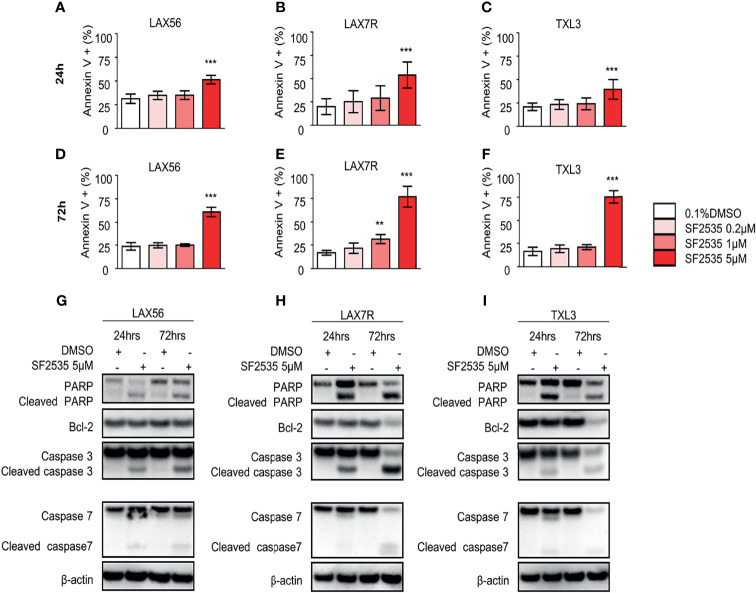
SF2535 induces apoptotic effects in B-ALL cells. LAX56, LAX7R and TXL3 B-ALL cells were cultured in presence of 0.1% DMSO (white bars) or SF2535 (0.2μM, 1μM, or 5μM) (in red bars) for 24 **(A–C)** and 72 hours **(D–F)** and apoptosis was assessed by percent of Annexin V^+^ cells by flow cytometry. Data are pooled from four independent experiments performed in triplicates. P-values are calculated using one-way ANOVA test and Tukey’s multiple comparison test. **P<0.01, ***P<0.001 compared to the DMSO group. LAX56, LAX7R and TXL3 B-ALL cells were cultured in presence of 0.1% or SF2535 5μM for 24 and 72 hours and proteins were isolated for Western blot analysis **(G–I)**. β-actin was used as loading control. One of two experiments is shown.

To determine the apoptotic effect of SF2535 in primary B-ALL, LAX56, LAX7R, and TXL3 cells were treated with DMSO or 0.2μM, 1μM, or 5μM SF2535 for 24 hours and 72 hours (**Figure 2**). The percentages of apoptotic cells were significantly increased after 24 hours with 5μM SF2535 compared to DMSO control for LAX56 (51.48 ± 4.51% vs 31.28 ± 4.94%, P<0.0001), LAX7R (53.86 ± 14.02% vs 19.98 ± 8.51%, P<0.0001), and TXL3 (39.51 ± 10.49% vs 20.90 ± 4.05%, P<0.0001) (**Figures 2A–C**), and were also significantly increased after 72 hours of treatment (**Figures 2D–F**) in four independent experiments performed. We further analyzed whether SF2535 affects components of the intrinsic apoptotic pathway, including caspase-3, caspase-7, PARP and the anti-apoptotic component BCL-2. 5μM SF2535 significantly increased cleaved PARP, caspase-3, and caspase-7 in LAX56, LAX7R, and TXL3. Moreover, SF2535 5μM markedly decreased BCL-2 after 72 hours in both LAX7R and TXL3, yet not in LAX56 (**Figures 2G–I**).

### SF2535 Causes Cell Cycle Changes and Suppresses Cell Counts in B-ALL

**Figure 3:**
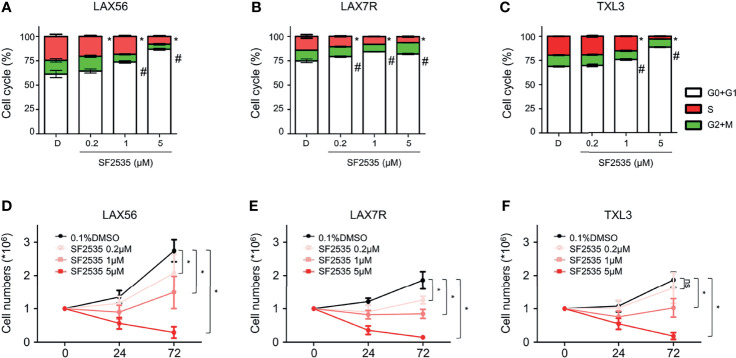
SF2535 prolongs G0+G1 phase arrest and attenuates S phase. **(A)** LAX56, **(B)** LAX7R and **(C)** TXL3 B-ALL cells were treatment with DMSO or SF2535 (0.2μM, 1 μM, or 5μM) for 24 hours. Cell cycle was assessed *via* flow cytometry after 24 hours of treatment. White bars indicate G0+G1 Phase. Green bars indicate G2+M Phase. Red bars indicate the S Phase. P-value *<0.05: Comparing S phase compared to DMSO S phase. P-value ^#^<0.05: Comparing G0+G1 phase to DMSO G0+G1 phase. The results are representative of two independent triplicate experiments. To determine the effect of SF2535 on proliferation and cell numbers, LAX56 **(D)**, LAX7R **(E)** and TXL3 **(F)** B-ALL cells were treated with DMSO or SF2535 (0.2μM, 1μM, or 5μM) for 24 and 72 hours. Numbers of live cells were counted by Trypan blue exclusion on a hemocytometer. Data of three independent triplicate experiments in triplicates are combined. P-value *<0.05 compared to DMSO; ns, not significant.

In order to determine if the decrease in proliferation was due to cell cycle arrest, we performed cell cycle analysis in SF2535-treated B-ALL cells. LAX56, LAX7R and TXL3 cells were treated with DMSO control or with 0.2μM, 1μM or 5μM of SF2535. After 24 hours, percentage of cells in G0+G1 phase increased while the percentage of cells in S phase decreased in all SF2535-treated groups except for TXL3 treated with 0.2μM of SF2535 (**Figure 3**). In LAX56 cells (**Figure 3A**), 0.2μM, 1μM and 5μM SF2535 when compared to DMSO prolonged G0+G1 phase (P=0.278, P=0.006, P<0.001, respectively) and arrested S phase (P=0.044, P=0.011, P<0.001, respectively). Similarly, significant prolonged G0+G1 phase was found in LAX7R (**Figure 3B**) and TXL3 (**Figure 3C**). The summarized results of mean and standard deviation of two independent triplicate experiments and representative flow cytometry figures are depicted in **Figure S4**. In addition, BrdU incorporation assays were performed, and similar results were shown (**Figure S5**). According to the apoptotic effect and S phase cell cycle arrest of SF2535, we performed cell count assays to assess the potential effects on proliferation by SF2535 in B-ALL cells. We treated B-ALL cells with DMSO or 0.2μM, 1μM, or 5μM SF2535 for 24 and 72 hours. Both 1μM and 5μM SF2535 significantly inhibited cell proliferation after 72 hours of treatment of LAX56 (**Figure 3D**), LAX7R (**Figure 3E**), and TXL3 (**Figure 3F**). For instance, at 72 hours, 1μM and 5μM of SF2535 reduced the number of viable cells compared to (2.74 ± 0.33) × 10^6^ in DMSO to (1.49 ± 0.49) × 10^6^in SF2535 1μM (P<0.0001) and (0.29 ± 0.17) × 10^6^ in SF2535 5μM (P<0.0001), in LAX56.

### SF2535 Decreases Surface Integrin Expression

**Figure 4:**
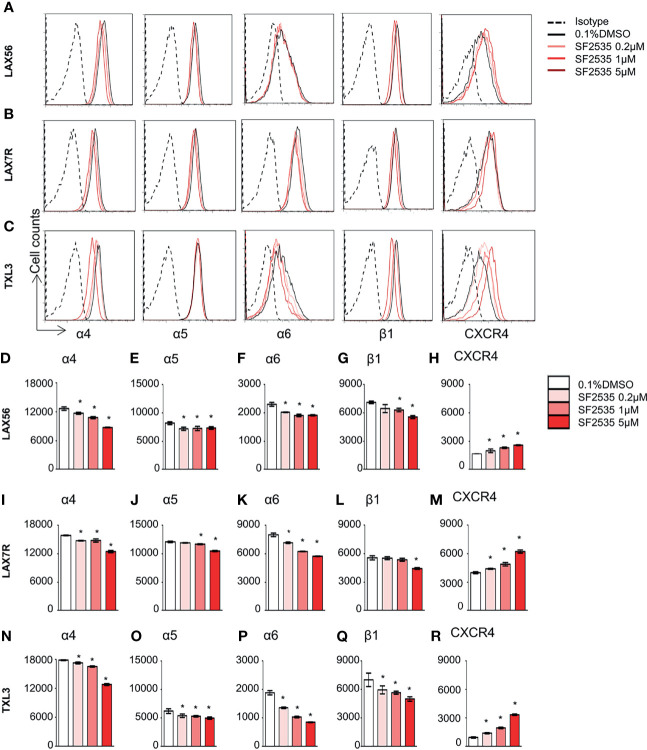
SF2535 affects adhesion molecules. Representative histograms for integrin α4, α5, α6, β1, and CXCR4 expression for **(A)** LAX56, **(B)** LAX7R and **(C)** TXL3 cells were treated with 0.1% DMSO (in white bars) or SF2535 (0.2μM, 1μM, 5μM in gradient red bars) for 24 hours. **(D–R)** Mean fluorescence intensity (MFI) of integrin α4, α5, α6, β1, and CXCR4 were shown on indicated y-axis in **(D–H)** LAX56, **(I–M)** LAX7R and **(N–R)** TXL3 B-ALL cells. P-value *<0.05 compared to DMSO control. One representative experiment out of at least two independent experiments is shown.

**Figure 5:**
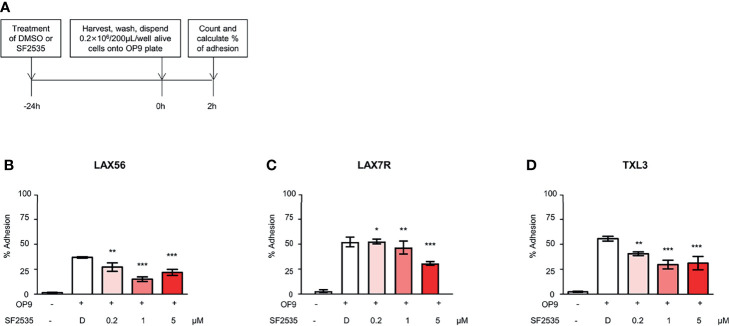
SF2535 moderately inhibits B-ALL adhesion to OP-9 cells. **(A)** Schema of adhesion assays. **(B)** LAX56, **(C)** LAX7R, and **(D)** TXL3 cells were treated with DMSO **(D)** or SF2535 for 24 hours. After harvest and wash, 0.2×10^6^ alive cells per well were placed on pre-seeded OP9 cells plate for 2 hours. Adhesion and supernatant cells were counted by Trypan Blue exclusion. Percentage (%) of alive adhesion cells were presented. Experiments were performed in triplicates. *P < 0.05, **P < 0.01, ***P < 0.001 compared to DMSO group.

Our previous studies have shown that cell adhesion-mediated drug resistance (CAM-DR) plays a crucial role in relapsed and refractory B-ALL (27, 28). Previously, we have shown inhibition of PI3Kδ with idelalisib in B-ALL inhibited homing of cells into the bone marrow (29). Decrease in homing may be due to the inability of cells to adhere to surrounding microenvironment upon PI3Kδ inhibition. In order to determine if blockade of PI3Kδ affects expression level of surface adhesion in B-ALL cells, we assessed integrin α4, α5, α6, β1, and CXCR4 expression in SF2535 treated B-ALL. LAX56, LAX7R and TXL3 were treated with DMSO control or with 0.2μM, 1μM or 5μM of SF2535. After 24 hours, cells were stained with anti-integrin α4, α5, α6, β1, and CXCR4 antibodies and their mean fluorescence intensity (MFI) was assessed by flow cytometry. To exclude dead cells which can interfere with flow cytometry data analysis, viable cells were strictly gated and a representative gating strategy of 5μM SF2535 treated LAX56 cells is shown in **Figure S6A**. As a result, histograms of integrin α4, α5, α6, β1, and CXCR4 showed relatively small changes in expression levels of integrin subunits and CXCR4 between DMSO and SF2535-treated groups (**Figures 4A–C**). Moreover, MFI of integrin α4, α5, α6, and β1 significantly decreased in SF2535 treated groups while MFI of CXCR4 increased in LAX56, LAX7R and TXL3 after SF2535 treatment (**Figures 4D–R**, **S6**). This result shows dual inhibition of PI3Kδ and BRD4 decreases integrin expression on the cell surface that is important for adhesion of leukemia cells to the microenvironment. However, SF2535 hardly decreased percentages of integrin α4, α5, and β1 expression (**Figures S6E–S**) and cells may compensate for the loss of integrin subunits by expressing other surface molecules implicated in adhesion, such as CXCR4. In order to further evaluate the physiological and biological relevance of integrin expression effect, we performed cell adhesion assays. Firstly, B-ALL cells were plated at 0.2 × 10^6^/200µL onto 96 well tissue culture plates seeded with or without irradiated OP9 stromal cells, which has multiple integrin ligands (25, 27), B-ALL cells were allowed to adhere for 4 hours. Subsequently, cells were treated with increasing SF2535 doses and cultured overnight. More de-adhered B-ALL cells were found in SF2535 treatment groups, whereas SF2535 induced more dead cells compared to DMSO vehicle control (**Figure S7**). Since SF2535 induces apoptosis of B-ALL, we pre-treated B-ALL with SF2535 for 24 hours and harvested live cells. Harvested cells were washed and plated at 0.2 × 10^6^/200µL onto 96 well tissue culture plates seeded with irradiated OP9 stromal cells for 2 hours allowing B-ALL cells to adhere. We observed that 5μM SF2535 significantly inhibited adhesion of the three B-ALL cells to stromal cells (**Figure 5**).

### SF2535 Decreases Peripheral Leukemic Burden in Mouse Model

We evaluated *in vivo* efficacy of the drug in a leukemia-engrafted mouse model. In order to determine the *in vivo* effects of SF2535, NSG mice were first intravenously injected with 1×10^6^ LAX56 cells per mouse. After 3 weeks of engraftment, the mice were treated either with the vehicle control (n=6) or SF2535 (30mg/kg, n=6) (**Figure S8A**). SF2535 was administered once to the mice and the early effects of the drug on B-ALL cells were evaluated. After 24 hours post-injection of SF2535, the mice were sacrificed and bone marrow (BM), spleen cells (SPC), and peripheral blood (PB) were collected and analyzed for human CD45^+^CD19^+^ expression *via* flow cytometry (**Figures S8B–D**). Leukemia burden, shown as percentage of human CD45^+^CD19^+^, in PB was significantly decreased in SF2535-treated mice (P=0.0202) (**Figure S8D**) yet there was no decrease of human leukemia in BM or SPC (**Figures S8B, C**). This result shows the dose and timing of SF2535 administration is sufficient to decrease leukemia burden in the peripheral blood. Effect of SF2535 on normal mature B-cells.

Finally, we tested SF2535 toxicity in two immortalized normal B cell lines, 3301015 and 5680001. At 24hours post-treatment, SF2535 decreased viability of 3301015 cells compared to control treated cells (71.6 ± 2.5%; N=3 vs 63.6 ± 0.3%; N=3) (P=0.03) while viability of 5680001 cells was not affected by SF2535. (**Figure S9**).

## Discussion

PI3K has been targeted by copanlisib and duvelisib which were approved by the FDA for use in CLL and follicular lymphoma (30, 31). It has been demonstrated that PI3K also plays a crucial role in ALL (4, 29). Our findings show that PI3K is broadly expressed in B-ALL and the key downstream signal p-AKT ^S473^ is markedly downregulated by dual inhibition of PI3Kδ-BRD4 by SF2535 (**Figure 1F**). In addition, it is well established that c-Myc plays a major role in mature B-ALL and Burkitt lymphoma (32, 33), however, there are few studies that explore the role of c-Myc in other types of B-ALL. Ott et al. reported that BET bromodomain inhibition using JQ1 targets both c-Myc and IL7R in high-risk *CRLF2*-rerranged and other B-ALL (34). Moreover, oncogenic Myc is also a difficult target for cancer therapy, and alternative approaches have been taken to indirectly target Myc by blocking pathway events upstream of c-Myc (12). Our previous study showed feasibility of targeting Myc with a dual-activity PI3K-BRD4 inhibitor (20). In our present study, we have demonstrated that c-Myc is expressed in B-ALL, and c-Myc was markedly downregulated by inhibition of its promoter site by SF2535 (**Figures 1B–D**). SF2535 also led to a decrease in p-AKT^S473^ levels upon inhibition of PI3Kδ. Our data show that SF2535 led to downregulation of both p-AKT and c-Myc in B-ALL. However, as BRD4 expression is highly variable in samples (**Figure 1A**, **S1B**), it would be appropriate to determine the BRD4 expression before SF2535 treatment.

Inhibition of c-Myc has been shown to result in apoptosis in T-ALL (34, 35). Our data also indicate that dual inhibition of PI3Kδ and BRD4 results in apoptosis or primary B-ALL cells using SF2535 (**Figures 2A–F**). Furthermore, we demonstrated that SF2535-induced apoptosis occurs through the intrinsic pathway *via* increasing cleavage of PARP, caspase-3 and caspase-7 and decreasing BCL-2 (36) (**Figures 2G–I**). Inhibition of PI3Kδ and BRD4 not only induced apoptosis, but also caused cell cycle arrest and decreased proliferation (**Figure 3**). A potential mechanism is that BET bromodomain inhibition affects key regulators of the cell cycle such as cyclin D1 expression (20).

Recently, we have suggested that BRD4 regulates the immunosuppressive myeloid tumor microenvironment which can be blocked by PI3K/BRD4 inhibitors using SF2523 (24). The bone marrow environment has been shown to promote CAM-DR in ALL (37). Our previous studies have identified the integrin α4 and α6 as an adhesion molecule that plays a critical role in B-ALL through CAM-DR (25, 27). Our results indicate dual inhibitors of PI3Kδ and BRD4 using SF2535 affected the expression of adhesion molecules including integrin α4, α5, α6, β1, while CXCR4 was increased (**Figure 4**). This finding suggests a relationship between integrins and PI3Kδ through outside-in signaling (28) and would warrant further mechanistic studies. It has been shown that integrin α6 and β1 are regulated by the c-Myc oncogene in colorectal cancer cells (38, 39) and a murine hematopoietic cell line (40). Yao et al. recently showed that use of a PI3Kδ inhibitor resulted in a significant reduction of leukemia metastasis to the central nervous system due to decreased integrin α6 expression despite minimally decreased bone marrow disease burden (41). It is possible that CXCR4 expression compensates for the downregulation of integrins, which requires further investigation. Although dual inhibitors of PI3K and BRD4 has been investigated in some solid tumors (20, 24), the current study is the first study to evaluate SF2535 in B-ALL. The bioavailability of SF2535 and its route of penetration and clearance remained unknown. Our preliminary *in vivo* results show that dual inhibition of PI3Kδ and BRD4 led to a reduction of leukemia cell numbers in the peripheral blood of leukemia bearing mice. Mice tolerated 10mg/kg SF2535 for continuous treatment up to 4 weeks, yet the low dosage of SF2535 was not effective enough to prolong the survival of leukemia engrafted mice, while higher doses 30 mg/kg were not well tolerated in mice (data not shown). We have shown in a small study that SF2535 may decrease viability of mature B-cells to a small but statistically significant extent. These results indicate that SF2535 may induce toxicity in normal B cells, which requires further follow-up studies.

Taken together, these results reveal that SF2535 efficaciously induces apoptosis through downregulating c-Myc and p-AKT pathways in primary B-ALL providing a rationale for further preclinical evaluation of PI3Kδ and BRD4 inhibition in B-ALL.

## Data Availability Statement

The original contributions presented in the study are included in the article/**Supplementary Material**. Further inquiries can be directed to the corresponding author.

## Ethics Statement

The animal study was reviewed and approved by IACUC CHLA. Written informed consent was obtained from the minor(s)’ legal guardian/next of kin for the publication of any potentially identifiable images or data included in this article.

## Author Contributions

Conceptualization of the study: DB, DD, and Y-MK. YR, HK, and Y-MK designed the research. YR, HK, HO, SH, EG, ZW, CN, NA-A, AC, Y-HL, CP, HA-A, DP, and SS performed the research, analyzed and interpreted the data. C-LH and ML contributed valuable material, technical expertise and interpreted the data. YR, HK, and Y-MK wrote the initial draft of the manuscript. All authors contributed to the article and approved the submitted version.

## Funding

This work was supported in part by the National Institutes of Health (R01 CA172896 to Y-MK and CA215656 to DD).

## Conflict of Interest

DD has ownership interest (including stock, patents, etc.) in and is a consultant/advisory board member of SignalRx Pharmaceuticals Inc.

The remaining authors declare that the research was conducted in the absence of any commercial or financial relationships that could be construed as a potential conflict of interest.

## Publisher’s Note

All claims expressed in this article are solely those of the authors and do not necessarily represent those of their affiliated organizations, or those of the publisher, the editors and the reviewers. Any product that may be evaluated in this article, or claim that may be made by its manufacturer, is not guaranteed or endorsed by the publisher.
