# Supplementary material for: Preclinical Evaluation of a Novel Dual Targeting PI3Kδ/BRD4 Inhibitor, SF2535, in B-Cell Acute Lymphoblastic Leukemia
Source: Front Oncol. 2021 Dec 1;11:766888. doi: 10.3389/fonc.2021.766888 (PMC8671162; doi:10.3389/fonc.2021.766888)
Supplement: Supplementary file 1 [file DataSheet_1.docx]

**Table S1. Characteristics of B-ALL**

| **Name** | **Clinical course** | **Cytogenetics** |
| --- | --- | --- |
| LAX7 | Diagnosis | Unknown |
| ICN24 | Diagnosis | Unknown |
| LAX53 | Diagnosis | Unknown |
| HEM14 | Diagnosis | Unknown |
| HEM18 | Diagnosis | IGH-CRLF2 |
| HEM127 | Relapse | P2RY8-CRLF2 |
| LAX7R | Relapse | IZKF1, KRAS^G12V^ |
| TXL3 | Diagnosis | BCR-ABL 1 |
| SFO2 | Diagnosis | BCR-ABL 1 |
| BLQ5 | Relapse | BCR-ABL 1 |
| HEM121 | Relapse | BCR-ABL 1 |
| Bel-1 | Cell line | MLL-rearranged |
| ICN13 | Diagnosis | MLL-rearranged |
| RS4;11 | Cell line | MLL-rearranged |
| Kasumi-2 | Cell line | TCF3-PBX1 |
| HEM31 | Diagnosis | ETV-RUNX1 |
| HEM35 | Diagnosis | ETV-RUNX1 |
| LAX56 | Relapse | t(Y;7) |
| LAX57 | Diagnosis | t(1;9) |

**Table S2. Western blot (WB) or chromatin immunoprecipitation (ChIP) antibody information**

| **Name of antibody** | **Dilution ratio** | **Company** |
| --- | --- | --- |
| anti-BRD4 | 1:1000 WB  1:50 ChIP | Cell Signaling |
| anti-PI3K p110δ | 1:1000 | Cell Signaling |
| anti-Phospho-Akt (Ser473) | 1:1000 | Cell Signaling |
| anti-Akt | 1:1000 | Cell Signaling |
| anti-c-Myc | 1:500 | Cell Signaling |
| anti-Caspase 3 | 1:1000 | Cell Signaling |
| anti-Caspase 7 | 1:1000 | Cell Signaling |
| anti-PARP | 1:1000 | Cell Signaling |
| anti-Bcl 2 | 1:1000 | Cell Signaling |
| anti-integrin α4 | 1:1000 | Cell Signaling |
| anti-integrin β1 | 1:1000 | Cell Signaling |
| anti-β-actin | 1:2000 | Santa Cruz Biotechnology |

**Table S3. Flow cytometry antibody information**

| **Name of antibody** | **Company** |
| --- | --- |
| anti-human CD19 FITC | BioLegend |
| anti-mouse CD45 FITC | BioLegend |
| anti-human CD45 APC | BioLegend |
| anti-human CD49d PE/Cy7 | BioLegend |
| anti-human CD49e PE | BioLegend |
| anti-mouse/human CD49f APC | BioLegend |
| anti-human CD184 PerCP/Cy5.5 | BioLegend |
| anti-human CD29 APC/Cy7 | BioLegend |
| Mouse IgG1, κ Isotype Ctrl Antibody FITC | BioLegend |
| Rat IgG2b, κ Isotype Ctrl Antibody FITC | BioLegend |
| Mouse IgG1, κ Isotype Ctrl Antibody APC | BioLegend |
| Mouse IgG1, κ Isotype Ctrl Antibody PE/Cy7 | BioLegend |
| Mouse IgG2b, κ Isotype Ctrl Antibody PE | BioLegend |
| Rat IgG2a, κ Isotype Ctrl Antibody APC | BioLegend |
| Mouse IgG2a, κ Isotype Ctrl Antibody PerCP/Cy5.5 | BioLegend |
| Mouse IgG1, κ Isotype Ctrl Antibody APC/Cy7 | BioLegend |
| CytoPhase™ Violet | BioLegend |
| Annexin V PE | BioLegend |
| DAPI | ThermoFisher |

**Figure S1. Corresponsive quantitative densitometric analysis of Figure 1A.** (A) ratio of PI3Kδ/β-actin expression, (B) ratio of BRD4/β-actin expression**.**

**Figure S2. EC50 curve and value of SF2535.** Percentage of apoptotic cells (Annexin V +) of (A, D) LAX56, (B, E) LAX7R and (C, F) TXL3 from treatment with DMSO, 0.04μM, 0.2μM, 0.5μM, 1μM, 5μM, or 10μM SF2535 for 48h were analyzed for the calculation of EC50. The value and 95% confidence intervals of EC50 of (A-C) were log-transformed. (D-F) show the percentages of apoptotic cells of DMSO and SF2535. Apoptosis assay was performing in triplicate using flow cytometry.

**Figure S3. Independent replicate experimental data supports downregulation of c-Myc and p-AKT by SF2535.** (A) and (B) c-Myc expression by Western Blot for Figure 1d and 1e. (C) Schema of phosphorylated AKT (Ser473) (p-AKT^S473^) detection assay. (D) p-AKT^S473^ expression by Western Blot for Figure 1f.

**Figure S4. SF2535 changes cell cycles. (A)** Representative cell cycle gating strategy, a sample from TXL3 with 5μM SF2535 treatment. Following treatment of DMSO or SF2535 (0.2μM, 1μM, 5μM) in **(B, E)** LAX56, **(C, F)** LAX7R, and **(D, G)** TXL3 for 24 hours, cell cycles were analyzed by flow cytometry. Representative histogram of cytophase were showed in **(B-D)**, while mean ± SD of two independent experiments showed in **(E-G)**.

**Figure S5. SF2535 prolongs G0+G1 phase arrest and attenuates S phase.** Following treatment of DMSO or SF2535 (0.2μM, 1μM, 5μM) in **(A, B)** LAX56, **(C, D)** LAX7R, and **(E, F)**TXL3 for 24 hour, cell cycles were analyzed by flow cytometry. **(B, D, F)** Representative dot plots of DMSO control and SF2535 5 μM were shown. Experiments were performed in triplicates. P-value *<0.05: Comparing S phase compared to DMSO S phase. P-value #<0.05: Comparing G0+G1 phase to DMSO G0+G1 phase.

**Figure S6. SF2535 affects adhesion molecules. (A)** Representative gating strategy for assessing adhesion molecules in LAX7R treated with SF2535 5μM. Representative dot plots for integrin α4, α5, α6, β1, and CXCR4 expression for **(B)** LAX56, **(C)** LAX7R and **(D)** TXL3 cells are treated with DMSO (in blank bars) or SF2535 (0.2μM, 1μM, 5μM in gradient red bars) for 24 hours. Percentages of integrin α4, α5, α6, β1, and CXCR4 expression for **(E-I)** LAX56, **(J-N)** LAX7R and **(O-S)** TXL3. P-value *<0.05. The results are the combination of at least two independent triplicate experiments.

**Figure S7. SF2535 moderately de-adheres B-ALL from stromal cell OP9.** (A) LAX56, (B)LAX7R, and (C)TXL3 cells were seeded with or without OP9 cells in 96-well plates for 4 hours. Subsequently, cells were treated with DMSO (D) or SF2535 (0.2 μM, 1 μM, 5 μM) and cultured for 16 hours. Percentages of alive adhesion cells were presented on upper panels of (A-C) while percentages of viability were shown on lower panels of (A-C). Dead cells were excluded by Trypan Blue counts. Experiments were performed in triplicates. * P<0.05, ** P<0.01, *** P<0.001, **** P<0.0001 compared to DMSO group.

**Figure S8. SF2535 decreases peripheral leukemia burden in primary B-ALL engrafted mice.** **(A)** Schematic for time flow of the experiment. 1×10^6^ cells (LAX56) were intravenously injected into NSG mice and were allowed to engraft for 3 weeks. Mice were treated with vehicle or SF2535 (30mg/kg) (n=6 each). After 24 hours post-treatment mice were sacrificed and bone marrow (BM), spleen (SPC), and peripheral blood (PB) were harvested. Percentages of human CD45 and human CD19 double positive primary B-ALL cells in **(B)** BM, **(C)** SPC, and **(D)** PB were assessed by flow cytometry after staining with anti-mouse CD45, anti-human CD45 and anti-human CD19. Ns, not significant. P-value *<0.05.

**Figure S9. SF2535 decreases viability of immortalized normal B cell line 3301015, but not in 5680001.** 0.5x10^6^ of immortalized normal B cell lines (A) 3301015 and (B) 5680001 were treated with 5μM of SF2535. After 24hours, cell viability was assessed using 7AAD and Annexin V staining via flow cytometry.
