# Supplementary figures and images for: Preclinical Evaluation of a Novel Dual Targeting PI3Kδ/BRD4 Inhibitor, SF2535, in B-Cell Acute Lymphoblastic Leukemia
Source: Front Oncol. 2021 Dec 1;11:766888. doi: 10.3389/fonc.2021.766888 (PMC8671162; doi:10.3389/fonc.2021.766888)

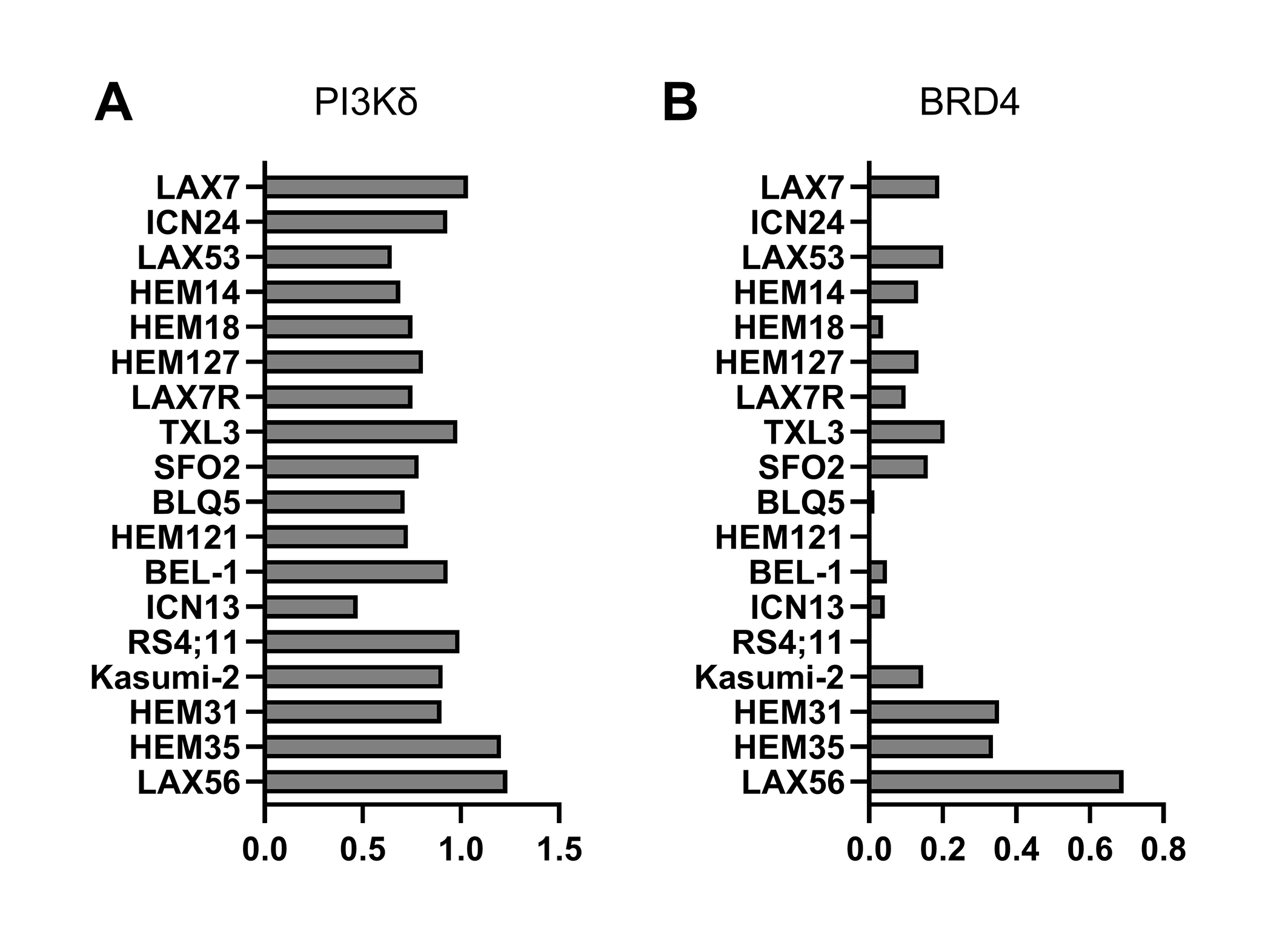

Supplement: Supplementary file 2 [file DataSheet_2.zip › Figure S1.JPEG]

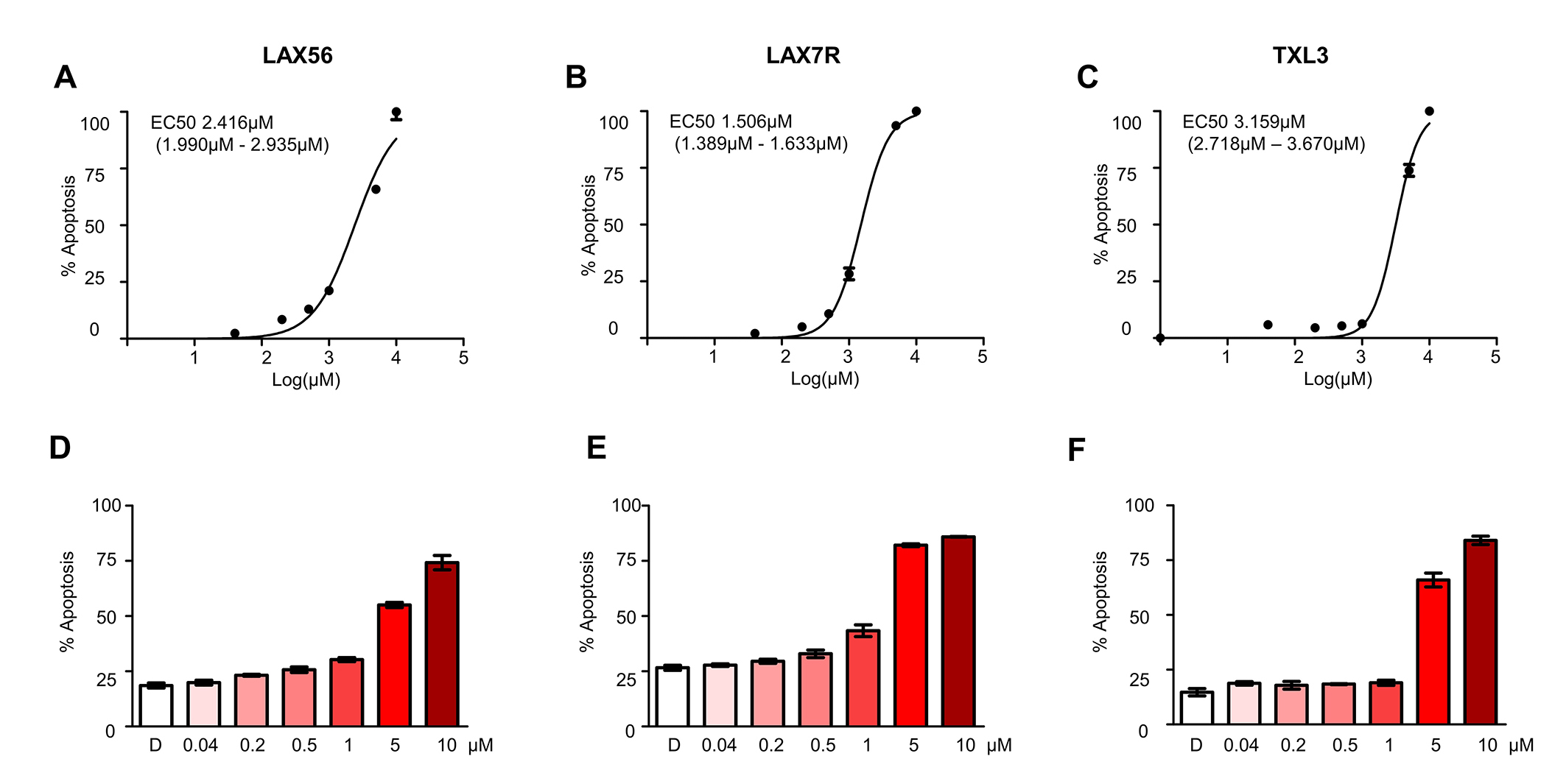

Supplement: Supplementary file 2 [file DataSheet_2.zip › Figure S2.JPEG]

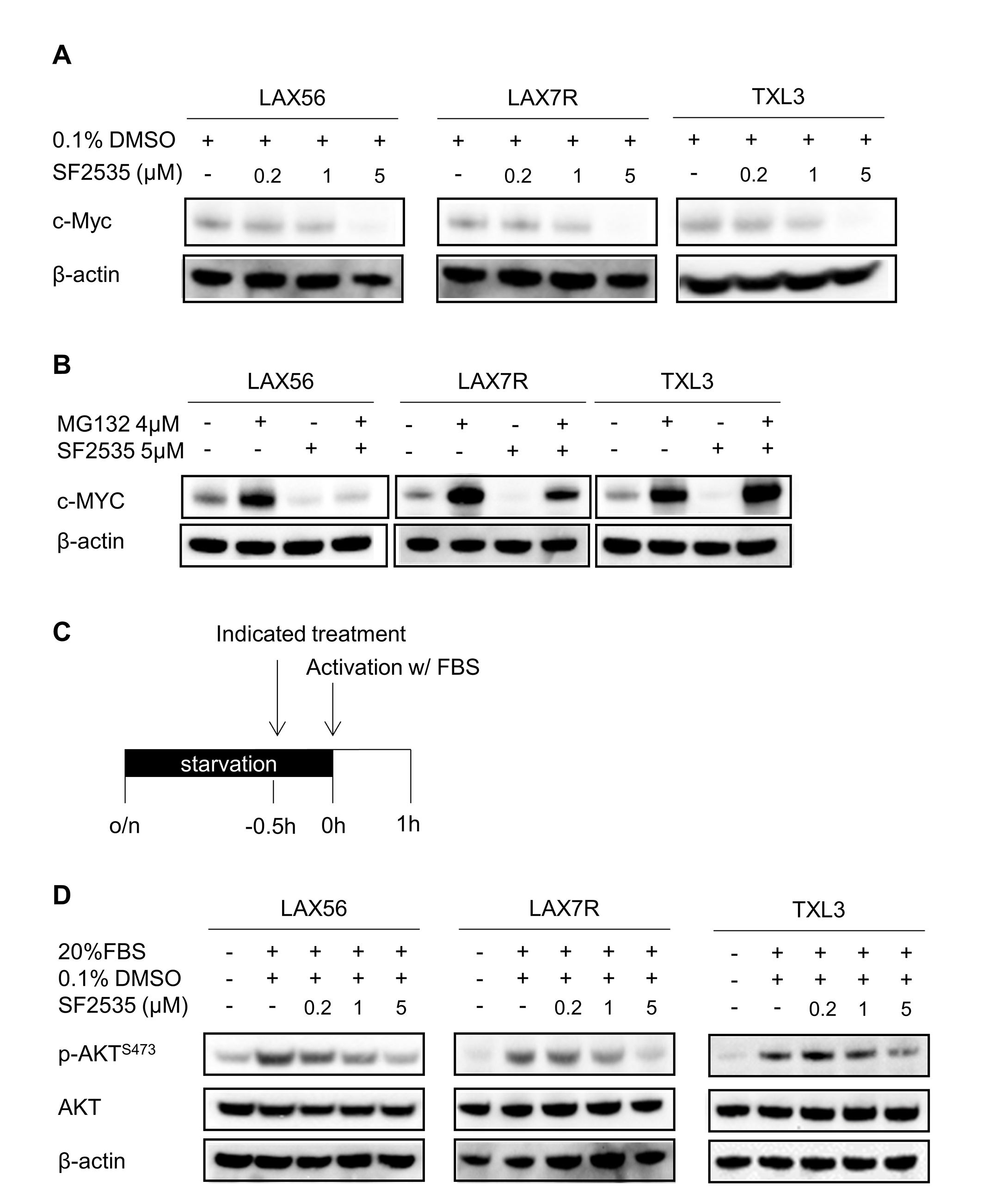

Supplement: Supplementary file 2 [file DataSheet_2.zip › Figure S3.JPEG]

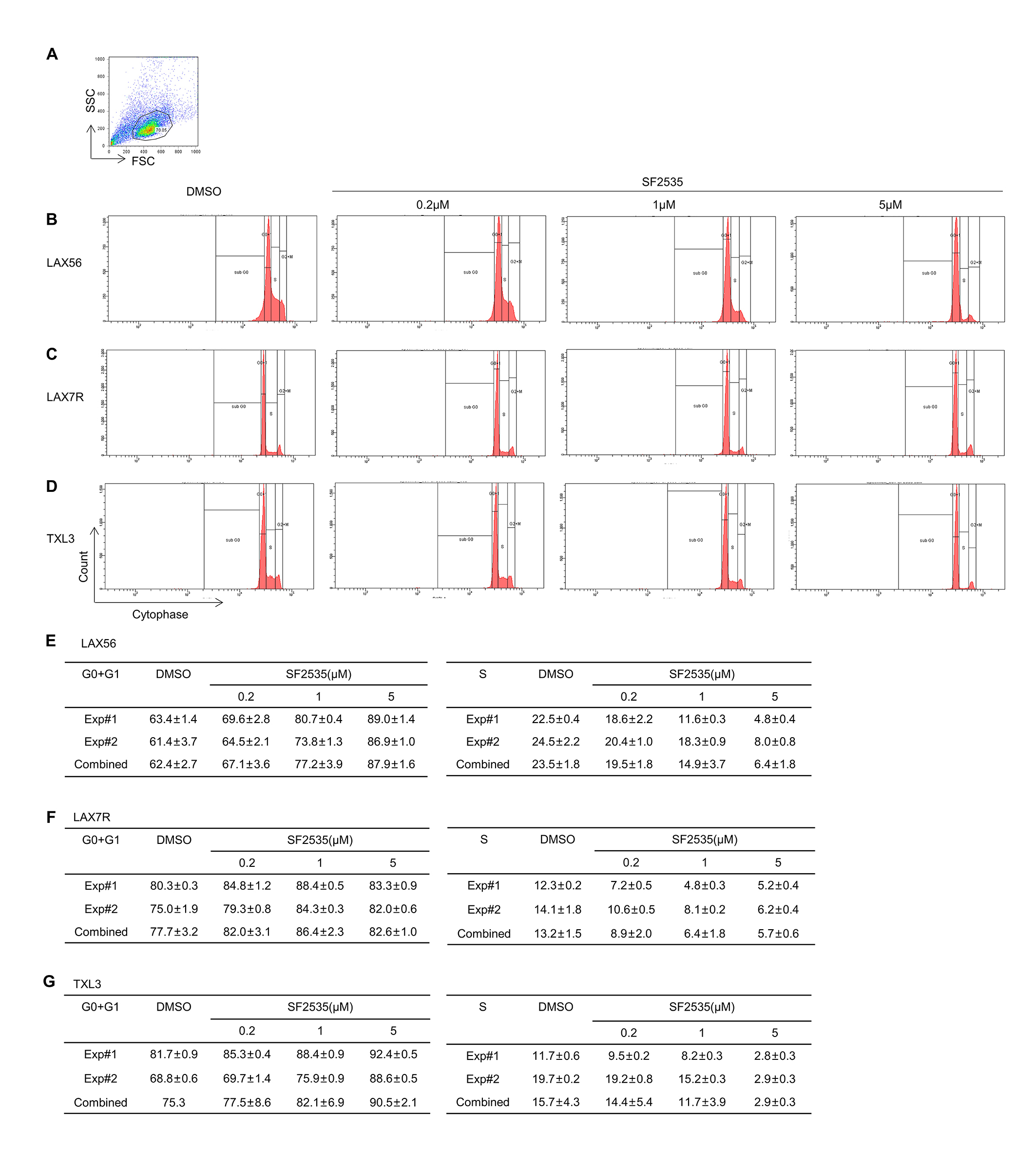

Supplement: Supplementary file 2 [file DataSheet_2.zip › Figure S4.JPEG]

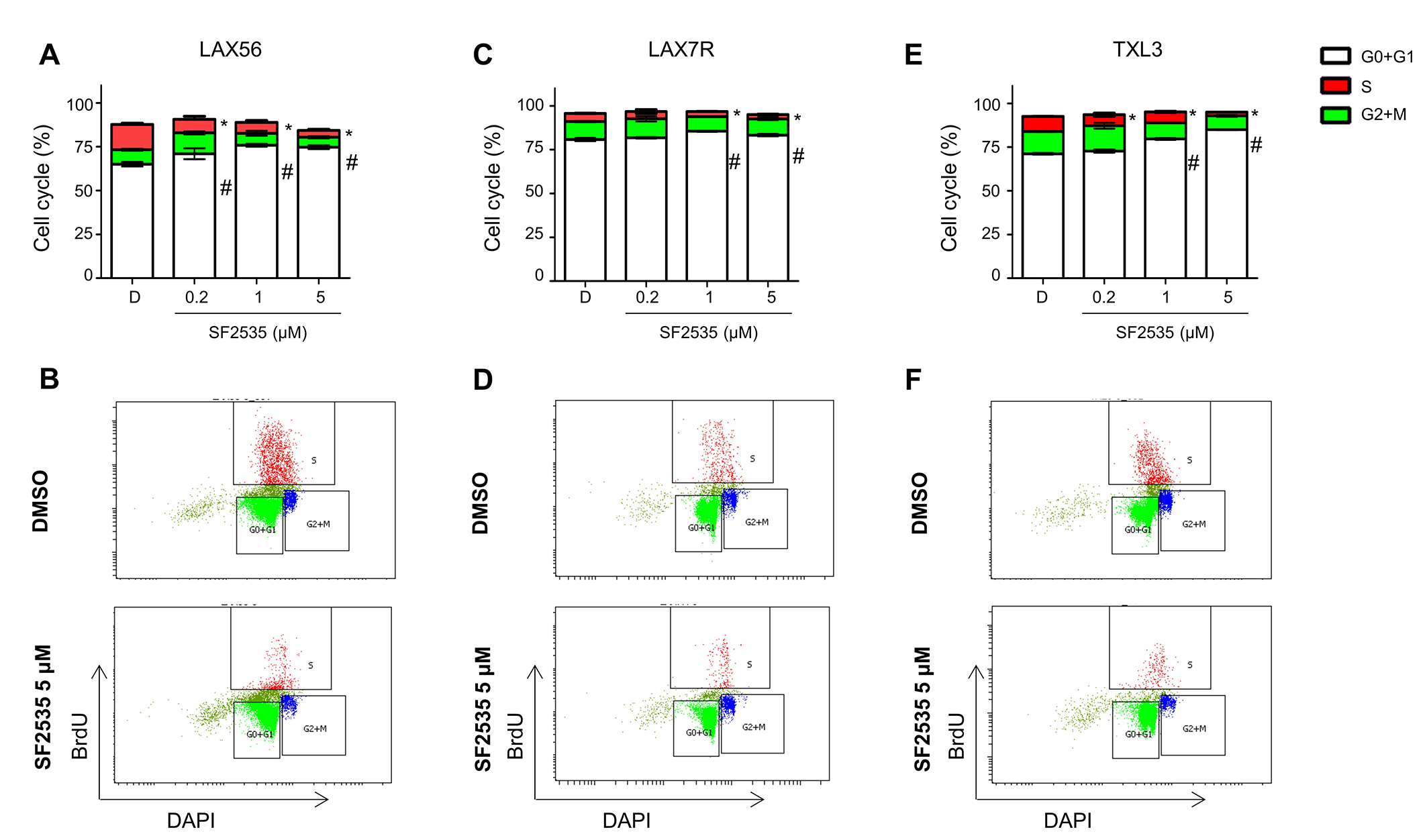

Supplement: Supplementary file 2 [file DataSheet_2.zip › Figure S5.JPEG]

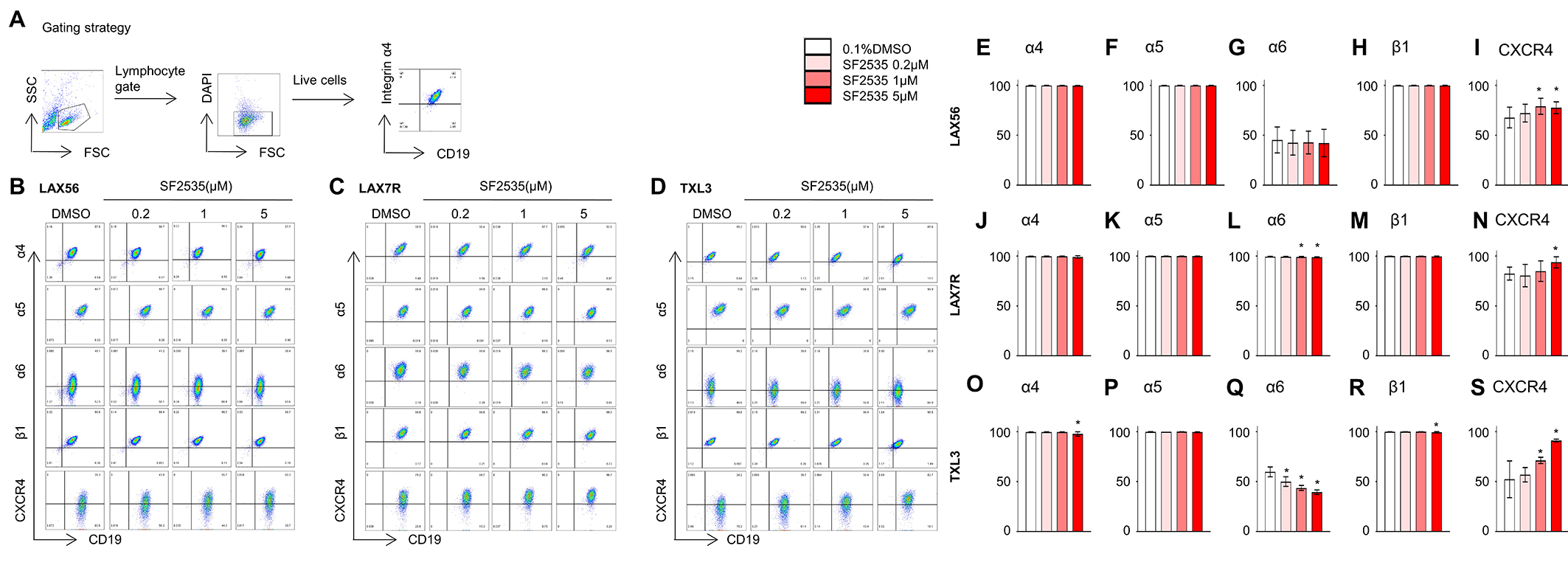

Supplement: Supplementary file 2 [file DataSheet_2.zip › Figure S6.JPEG]

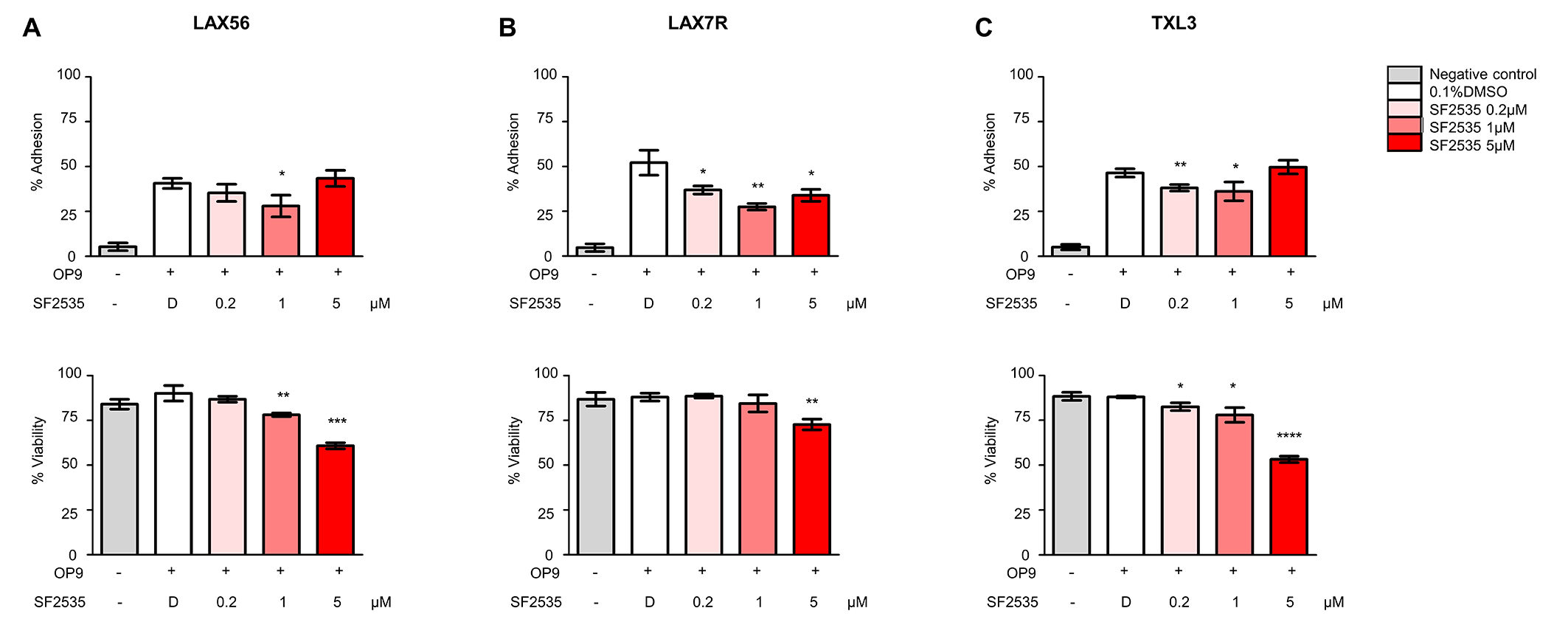

Supplement: Supplementary file 2 [file DataSheet_2.zip › Figure S7.JPEG]

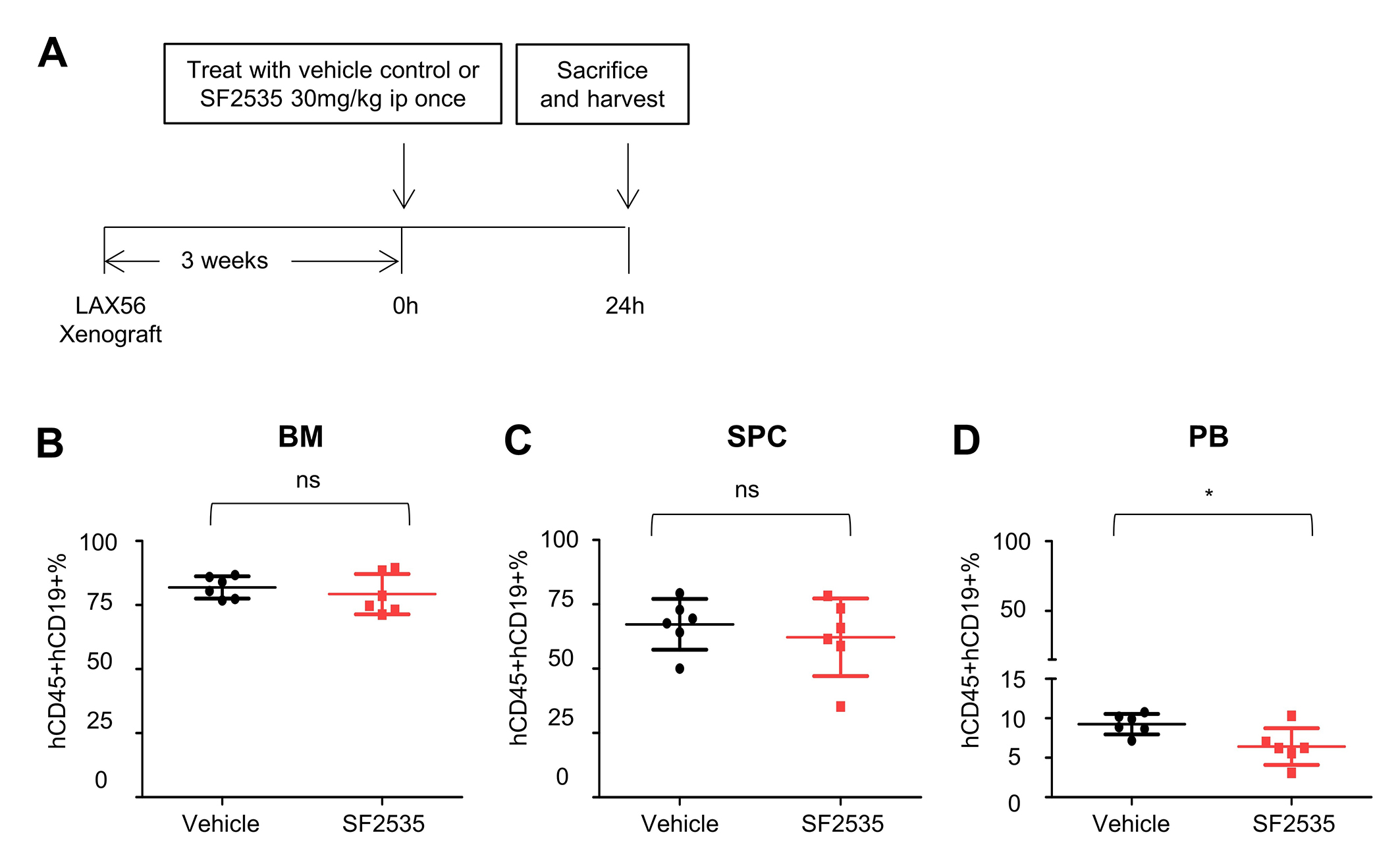

Supplement: Supplementary file 2 [file DataSheet_2.zip › Figure S8.JPEG]

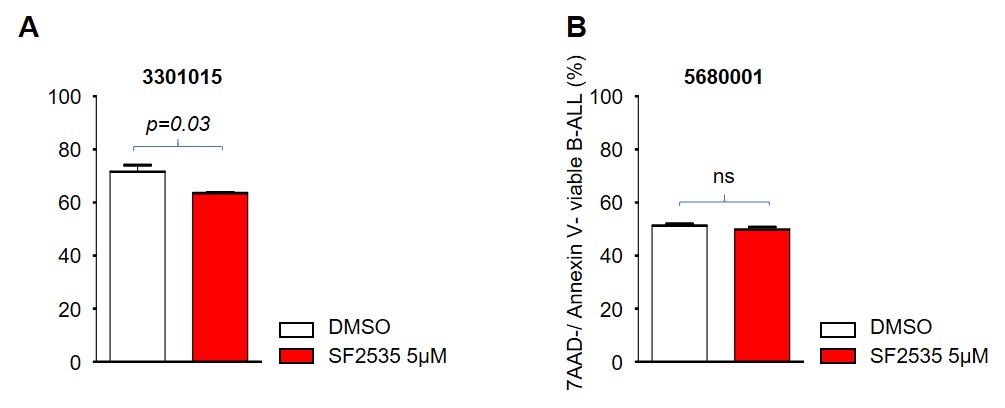

Supplement: Supplementary file 2 [file DataSheet_2.zip › Figure S9.JPEG]
